# High NT-proBNP level is correlated with high PEEP, low PH and low PaO_2_/FiO_2 _in ARDS

**DOI:** 10.1186/cc10717

**Published:** 2012-03-20

**Authors:** Y Nassar, D Monsef, S Abdelshafy, G Hamed

**Affiliations:** 1 Cairo University, Giza, Egypt

## Introduction

Cardiac injury may occur in ARDS patients with structurally normal hearts and may be correlated with respiratory parameters [1]. We aimed at observing NT-proBNP, troponin I and troponin T relations with different respiratory parameters in ARDS.

## Methods

Inclusion criteria were any adult patient diagnosed to have ARDS according to the criteria of the American-European Consensus Conference in 1994. Exclusion criteria were any structural heart disease by echo, pulmonary embolism, atrial fibrillation, renal insufficiency, and age <18. All patients benefited from a lung protective ventilation strategy. Plasma NT-proBNP, troponin I and troponin T were measured on day 0 and on day 2 and day 7 of ARDS diagnosis. PH, PaCO_2_, PaO_2_, P(A-a)O_2 _(alveolar-arterial gradient), PaO_2_/FiO_2 _ratio, a/A ratio, PEEP, PIP (peak airway pressure), P_mean_, P_plat _(plateau pressure) and C_eff _(effective compliance), and R_aw _(airway resistance) were monitored daily.

## Results

The study comprised 20 patients with mean age of 58.9 ± 20.69 years, 11 men versus nine women (*P *>0.05). NT-proBNP was negatively correlated with PH on day 2 (*P = *0.008, *r *= -0.53) and day 7 with (*P = *0.02, *r *= -0.50). NT-proBNP was positively correlated with PEEP on day 2 (*P = *0.05, *r *= 0.46) and day 7 (*P = *0.035, *r *= 0.48). NT-proBNP was negatively correlated with the PaO_2_/FiO_2 _ratio on day 7 (*P = *0.0035, *r *= 0.60). However, there was no significant correlation between NT-proBNP and other respiratory indices including PaCO_2_, HCO_3_, PaO_2_, SaO_2_, FiO_2_, PAO_2_, P(A-a)O_2 _and a/A ratio (*P *>0.05). Neither troponin I nor troponin T showed any significant correlation with any respiratory indices PH, PEEP, PaO_2_/FiO_2_, PaCO_2_, HCO3, PaO_2_, SaO_2_, FiO_2_, PAO_2_, P(A-a) O_2 _and a/A ratio on any day (*P *>0.05). None of the cardiac markers NT-proBNP, troponin I or troponin T showed any significant correlation with the lung mechanics parameters C_dyn_, R_aw_, C_eff_, PIP, P_plat_, and P_mean _(*P *>0.05).

## Conclusion

High NT-proBNP level was correlated with high PEEP, low PH and low PaO_2_/FiO_2 _ratio while troponin T and troponin I did not show significant correlations with respiratory parameters in ARDS patients with structurally normal hearts.
